# Multiple functions of the herpesvirus UL14 gene product in viral infection

**DOI:** 10.3389/fmicb.2024.1483022

**Published:** 2024-10-23

**Authors:** Jieyu Wan, Mingshu Wang, Anchun Cheng, Wei Zhang, Qiao Yang, Bin Tian, Xumin Ou, Di Sun, Yu He, Xinxin Zhao, Ying Wu, Shaqiu Zhang, Juan Huang, Zhen Wu, Yanling Yu, Ling Zhang, Dekang Zhu, Mafeng Liu, Shun Chen, Renyong Jia

**Affiliations:** ^1^Institute of Veterinary Medicine and Immunology, Sichuan Agricultural University, Chengdu, China; ^2^Sichuan Agricultural University, Key Laboratory of Animal Disease and Human Health of Sichuan Province, Chengdu, China; ^3^Sichuan Agricultural University, International Joint Research Center for Animal Disease Prevention and Control of Sichuan Province, Chengdu, China; ^4^Engineering Research Center of Southwest Animal Disease Prevention and Control Technology, Ministry of Education of the People's Republic of China, Chengdu, China; ^5^Research Center of Avian Disease, College of Veterinary Medicine, Sichuan Agricultural University, Chengdu, China; ^6^Sinopharm Yangzhou VAC Biological Engineering Co., Ltd., Yangzhou, China

**Keywords:** herpesvirus, pUL14, tegument proteins, viral replication, viral infection

## Abstract

Herpesviruses are a family of double-stranded DNA viruses with a tegument structure and a genome composed of a single sequence and terminal repeat (TR) sequences. The herpesvirus UL14 gene encodes the protein UL14 (pUL14), which has various subcellular localizations and plays a vital role in regulating immediate–early (IE) gene transcription and expression, influences the intracellular localization patterns of several proteins belonging to the capsid and the DNA packaging machinery, participates in secondary envelopment, and influences viral particle release. Additionally, pUL14 has roles in maintaining cellular homeostasis and preventing apoptosis. This review discusses how pUL14 engages in the life cycle of herpesviruses and provides new ideas for further research on pUL14’s function in viral infection.

## 1 Introduction

**Figure 1:**
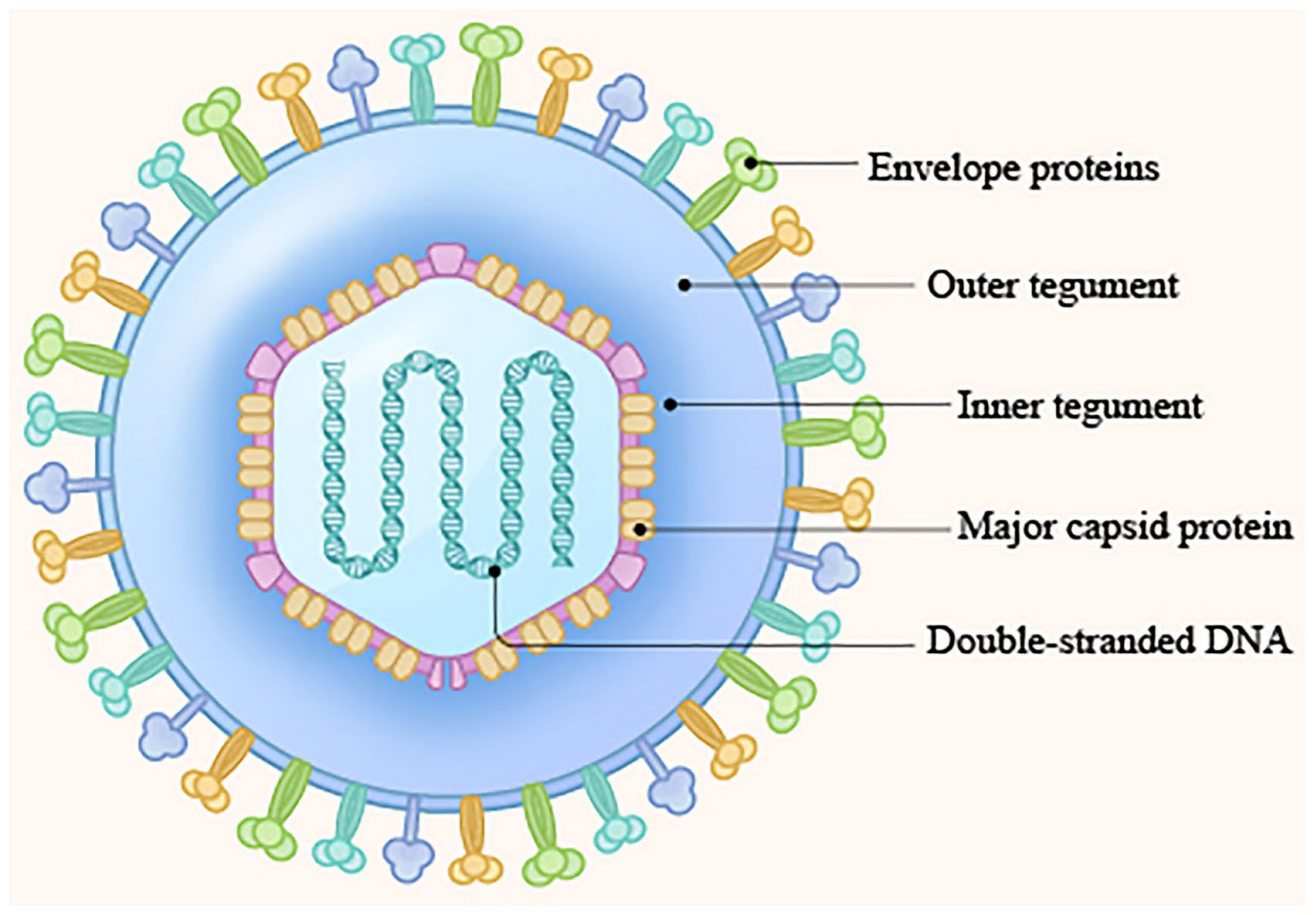
Structure of the herpesvirus genome. (1) Double-stranded DNA genome; (2) Capsid protein; (3) Tegument protein; (4) Envelope protein (Boehmer and Lehman, 1997; Hulo et al., 2011).

The *Herpesviridae* is a family of large double-stranded DNA-enveloped viruses (Connolly et al., 2021) that includes many important human and animal pathogens. At present, herpes simplex virus 1 (HSV-1; Xu et al., 2023), herpes simplex virus 2 (HSV-2; Truffot et al., 2023), varicella-zoster virus (VZV; Bloch et al., 2023), Epstein–Barr virus (EBV; Li et al., 2023), human cytomegalovirus (HCMV; Nuévalos et al., 2023), and human herpesvirus 6, 7, and 8 (HHV-6, HHV-7, and HHV-8; Hamada et al., 2023; Naipauer and Mesri, 2023) are associated with human diseases. Animal herpesviruses include bovine herpes virus type 1 (BoHV-1; Jones, 2019), pseudorabies virus (PRV; Pomeranz et al., 2005), Marek’s disease virus (MDV; Teng et al., 2023) and duck plague virus (DPV), which primarily cause diseases in animals (Shen et al., 2023). On the basis of differences in genomic homology, host range of infection, replication efficiency, latent cell species, and pathogenicity, the herpesvirus family is divided into the *Alphaherpesvirinae* (α), *Betaherpesvirinae* (β), and *Gammaherpesvirinae* (γ) subfamilies by the International Commission on Taxonomy of Viruses (ICTV; Whitley et al., 1998; Gatherer et al., 2021). HSV-1, HSV-2, VZV, BoHV-1, PRV, MDV, and DPV are members of the alphaherpesvirus subfamily. HCMV, HHV-6, and HHV-7 belong to the betaherpesvirus subfamily, and HHV-8 and EBV belong to the gammaherpesvirus subfamily. The virion is comprised, from inside to outside, of four layers: the double-stranded DNA genome, capsid, tegument, and envelope (Boehmer and Lehman, 1997; Hulo et al., 2011; Figure 1). The tegument is a unique structure of herpesviruses that plays critical roles in the life cycle of the virus (Compans et al., 2008), such as regulating kinase activity, modulating innate immunity, and modulating viral gene transcription and expression (Burgess and Mohr, 2018; Van Gent et al., 2022; Sanders et al., 2023). In addition, the tegument protein, as a structural protein of the virion, connects the capsid with the envelope and plays a key role in the process of virus packaging, maturation, and release (Thomas et al., 2022; Uddin et al., 2023; Wu et al., 2023). At present, studies on the tegument have focused mainly on viral gene transcriptional regulatory proteins with specific molecular biological significance and functional proteins with specific effects on cells, such as VP22 (Pheasant et al., 2023) and VP16 (Ma et al., 2023). In contrast, studies on small tegument proteins, such as pUL14, are relatively rare. However, this protein also plays an important role in regulating immediate–early (IE) gene transcription and expression, influencing the intracellular localization patterns of a number of proteins belonging to the capsid and the DNA packaging machinery, participating in secondary envelopment, and influencing viral particle release. In this review, we summarize the general structural features and functions of pUL14 and how pUL14 participates in the life cycle of herpesviruses and provide new ideas for further research on pUL14’s function in viral infection.

## 2 Characteristics of the herpesvirus UL14 gene

### 2.1 The herpesvirus UL14 gene is located on the viral genome

**Figure 2:**
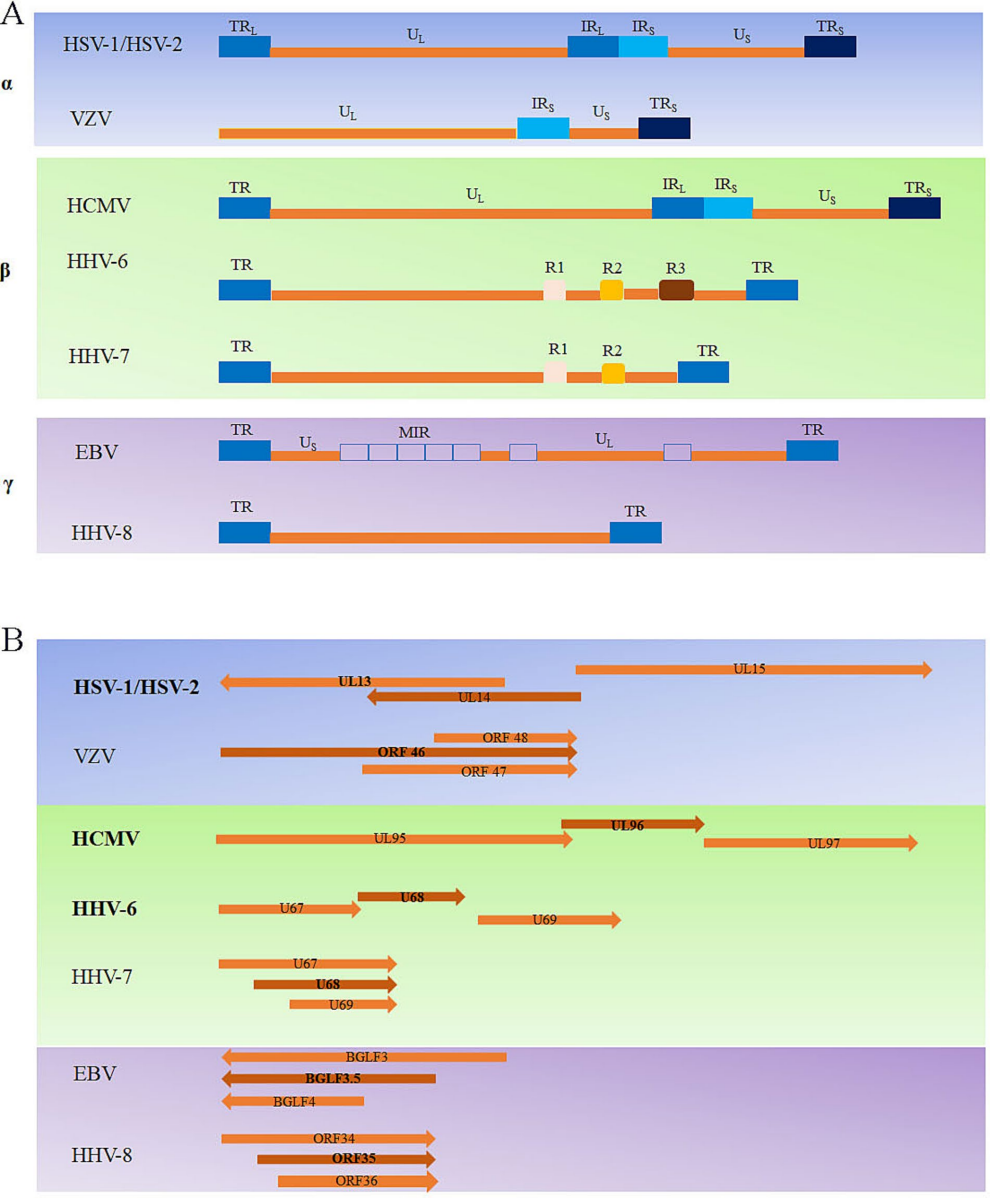
Overview of the types of herpesvirus genomes **(A)** and the herpesvirus UL14 genes located in the genome **(B)**.

The arrangement of reiterated sequences in the herpesvirus genome, whether as direct or inverted repeats at the genome termini or internally, leads to the formation of various classes of genome architecture. Some of these classes include orientation isomers resulting from recombination between terminal and internal inverted repeats. The type A genome, represented by HHV-6/7 (Berneman et al., 1992; Braun et al., 1997; Dominguez et al., 1999), has a unique sequence flanked by terminal repeat (TR) sequences on both sides, and the HHV-8 genome (Russo et al., 1996) has a similar arrangement. Type C genomes, represented by the EBV (Baer et al., 1984) genome, have a sequence of the unique long (UL) component and a sequence of the unique short (US) component, separated by a middle internal repeat (MIR). In the D-type genomes, as represented by VZV (Scott and Davison, 1986), a TR sequence undergoes an internal inversion, and the US surrounded by the inverted repeat sequence can invert relative to the UL. The E-type viral genomes, represented by the HSV-1/2 (McGeoch et al., 1988; Dolan et al., 1998) and HCMV genomes (Akrigg et al., 1985; Dunn et al., 2003; Rigoutsos et al., 2003), are composed of UL, US, TR, and internal repeat (IR) sequences (Figure 2A).

The UL14 gene is conserved in the alphaherpesviruses, and the coding region overlaps that of UL13, which encodes a protein kinase (Cunningham et al., 2000). The homologous genes in other herpesviruses include VZV ORF46, HCMV UL96, HHV-6 U68, HHV-7 U68, Kaposi’s sarcoma-associated herpesvirus (KSHV) ORF35, and EBV BGLF3.5. The position of the UL14 gene in the genome varies among the different human herpesviruses: HHV-6/7 U68 and KSHV ORF35 are unique sequences in the middle of the genome, and the rest are located in the UL region. HSV-1/2 UL14, HCMV UL96, and HHV-6 U68 partially overlap with the neighboring genes UL13, UL95, and U67 on one side. However, VZV ORF46, HHV-7 U68, EBV BGLF3.5, and KSHV ORF35 partially overlap with neighboring genes (Figure 2B). Overall, owing to the large differences in genome arrangement among members of the α-herpesvirus, β-herpesvirus, and γ-herpesvirus subfamilies, there are also differences in the arrangement of the genes homologous to the UL14 gene in these three subfamilies.

### 2.2 The types of herpesviruses UL14 genes

Herpesvirus genes are transcribed by the cell’s DNA-dependent RNA transcriptase. The genes fall into three main transcription regulation classes: IE, early genes (E), and late genes (L), of which late genes can be divided into early late genes (partly dependent on the synthesis of viral DNA) and late genes (highly dependent on the synthesis of viral DNA; Subak-Sharpe and Dargan, 1998). The IE gene is the first to be transcribed and the first to be expressed. It initiates and guides the transcription and expression of early and late genes. The early genes are transcribed before viral DNA replication after IE protein activation and reach the highest transcription levels after replication begins. Finally, the late genes, which encode mainly structural proteins of the virus, are expressed (Honess and Roizman, 1974). HSV-1/2 pUL14 is expressed late in infection and is considered a late gene (Cunningham et al., 2000; Yamauchi et al., 2001). However, HCMV UL96 is an early gene (Tandon and Mocarski, 2011). These findings suggest that the UL14 gene in various herpesvirus subfamilies exhibits different gene transcription patterns, implying that its functions might also vary.

## 3 Characteristics of the protein encoded by the herpesvirus UL14 gene

### 3.1 The molecular weight of the protein encoded by the UL14 gene

The herpesvirus UL14 gene encodes a relatively small protein (Table 1), and phosphorylation results in different molecular masses of pUL14. HSV-1/2 pUL14 is predicted to be a 219 amino acid protein with a molecular mass of 23 kDa.

However, in HSV-1-infected rabbit skin fibroblast (RS) cells, the UL14 gene product was identified as a 32 kDa protein, whereas in HSV-2-infected Vero cell lysates, 34, 33, and 28 kDa proteins were detected. Cunningham C et al. speculated that these differences might result from phosphorylation based on their phosphatase treatment and immunoblotting experiments (Wada et al., 1999; Cunningham et al., 2000). Additionally, in HEK293T cells transfected with MHV-68 ORF35 (the gene homologous to HSV-1 UL14), 26 and 28 kDa proteins were detected, which was attributed to phosphorylation at S124, S126, and S129 (Hikita et al., 2015). In summary, the size of pUL14 varies among herpesviruses.

**Table 1:** Characterization of the herpesvirus UL14 gene and its homologous proteins.

Virus	Gene	Number of amino acids (aa)	Protein molecular weight prediction/detection (kDa)
HSV-1	UL14	219	23.9/32 (Cunningham et al., 2000)
HSV-2	UL14	219	23.9/28, 33, 34(Wada et al., 1999)
DPV	UL14	154	17.5/17.5 (Li et al., 2016)
HCMV	UL96	127	14.4/14 (Tandon and Mocarski, 2011)
EBV	BGLF3.5	154	17.5/17.5 (Watanabe et al., 2015)
KSHV	ORF35	149	16.8/16.8 (Bergson et al., 2016)
MHV-68	ORF35	152	17.1/26, 28 (Hikita et al., 2015)

DPV, duck plague virus; EBV, Epstein–Barr virus; KSHV, Kaposi’s sarcoma-associated herpesvirus; HCMV, human cytomegalovirus; HSV-1, herpes simplex virus 1; HSV-2, herpes simplex virus 2.

### 3.2 Localization of the protein encoded by the UL14 gene

#### 3.2.1 Subcellular localization of pUL14

The most common subcellular localizations of proteins are whole-cell localization, cytoplasmic localization, nuclear localization, and nucleolar localization. The subcellular localization of a protein is crucial for understanding its function (Nair, 2003). For viruses, the varying subcellular localizations of their proteins indicate distinct functions. After synthesis in the cytoplasm, viral proteins are transported to specific locations where they can effectively carry out their functions (Xu and Massagué, 2004; Terry et al., 2007; Liu et al., 2016; Wubben et al., 2020; Li J. et al., 2021; Wing et al., 2022). The herpesvirus viral DNA genome replicates in the nucleus and assembles with the capsid protein to form a nucleocapsid (Johnson and Baines, 2011; DeRussy et al., 2016). The nucleocapsid moves to the inner nuclear membrane for primary envelopment (Mettenleiter et al., 2013; Takeshima et al., 2019). The nucleocapsid escapes from the nucleus, enters the cytoplasm, and undergoes secondary envelopment (Owen et al., 2015). Therefore, their localization can preliminarily predict the possible functions of viral proteins in the cell.

pUL14 shows different intracellular distribution patterns when expressed alone. Indirect immunofluorescence assay (IFA) results revealed that the HCMV UL96 (Tandon and Mocarski, 2011), MHV-68 ORF35 (Hikita et al., 2015), and KSHV ORF35 (Masa et al., 2008) gene products were localized only in the cytoplasm and were not affected by other viral proteins. However, the subcellular localization of alphaherpesvirus pUL14 varies and includes whole-cell, cytoplasmic, and nuclear localization. In transfected cells, this diversity in localization was observed simultaneously at the same time. For example, HSV-2 pUL14, when expressed alone, exhibited a varied intracellular distribution 24 h after transfection: it localized to the cytoplasm in 70% of the expressing cells, was found in both the cytoplasm and nucleus in 15% of the expressing cells, and was exclusively nuclear in 15% of the expressing cells (Yamauchi et al., 2001). Additionally, the distribution of pUL14 in the cells is dynamic: in DPV-infected duck embryo fibroblasts (DEFs), pUL14 was mainly distributed in the cytoplasm in the early stages of viral infection but migrated to the nucleus in the late stage of infection (Li et al., 2016). Currently, there is no research indicating that HSV-1/2 pUL14 has a nuclear localization signal (NLS); however, a region that is highly conserved between HSV-1 and HSV-2, especially ^60^RLKSRARLE^68^, is similar to an α-helix contained within the substrate-binding region of Hsp70. One study showed that pUL14 translocates to the nucleus and nucleolus upon stress, such as heat shock and osmotic shock (Yamauchi et al., 2002a, 2002b, 2003). In addition, when pUL14 was cotransfected with other viral proteins, such as VP16, VP26, and UL17, it not only facilitated the nuclear localization of these other viral proteins but also enhanced their nuclear localization ability; furthermore, these proteins colocalized within the nucleus. Notably, a bimolecular fluorescence complementation (BiFC) assay was used to confirm a direct interaction between DPV pUL14 and VP16 (Yamauchi et al., 2001; Ohta et al., 2011). In summary, pULl4 displays a variety of modes of subcellular localization through the nuclear membrane and is regulated by various factors, which also suggests that pUL14 may have multiple functions.

#### 3.2.2 Location of the UL14 protein in the virus structure

To determine whether pUL14 is a component of virion particles and what its location is in virion particles, mass spectrometry and Western blotting were used, which revealed that HSV-1 and HSV-2 pUL14 are minor components of the virion tegument (Cunningham et al., 2000; Yamauchi et al., 2001). Kerstin Radtke further identified HSV-1 pUL14 as an inner tegument protein (Radtke et al., 2010). The localization of pUL14 in the structure of other herpesviruses has not yet been reported. Overall, the results indicate that HSV-1/2 pUL14 is a tegument protein.

## 4 Function of herpesvirus pUL14

### 4.1 Role of pUL14 in virus replication

**Figure 3:**
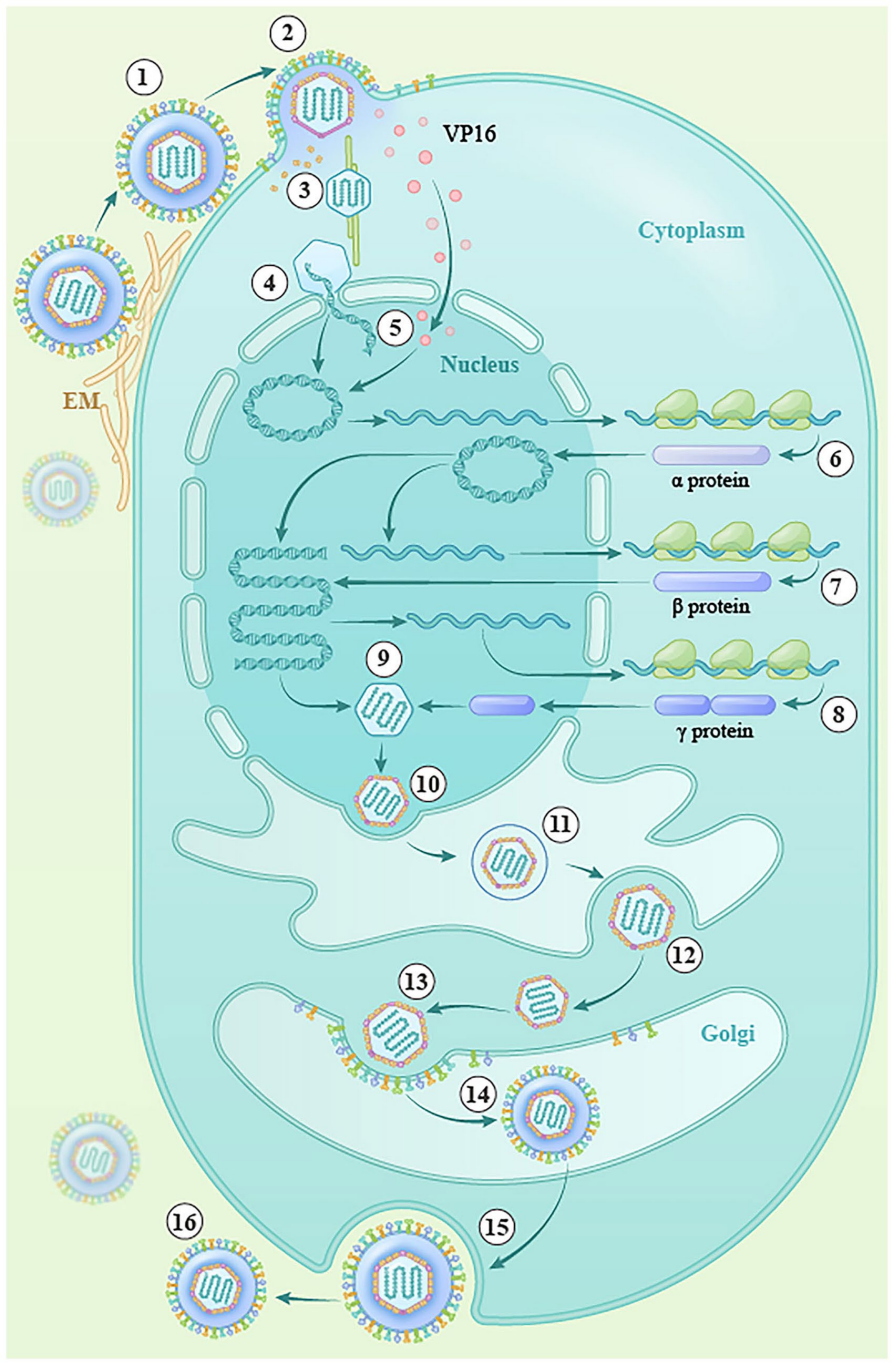
The lytic replication cycle of herpesviruses. (1) Entry into the cell (steps ①, ②), (2) transfer of the capsid to the nucleus (steps③–⑤), (3) viral DNA replication and transcription (steps ⑥–⑧), (4) capsid assembly (step ⑨), (5) egress from the nucleus (steps ⑩–⑫), (6) maturation and envelopment of viral particles in the cytoplasm (steps ⑬, ⑭), and (7) release of mature virions (steps ⑮, ⑯).

Herpesvirus proliferation mainly includes entry, replication, virion assembly, primary and secondary envelopment, and release of mature virions (Demangone et al., 1987; Boehmer and Lehman, 1997; Figure 3). The herpes simplex virus enters cells through the action of four glycoproteins, gB, gD, gH, and gL, and the viral nucleocapsid is then released into the cytoplasm (steps 1 and 2; Gerber et al., 1995; Herold et al., 1996; Cheshenko and Herold, 2002; Atanasiu et al., 2007; Gianni et al., 2009; Connolly et al., 2011; Karasneh and Shukla, 2011). After entering the cell, most viral tegument proteins are dissociated by the actions of pUS3 and pUL13, and the remaining few tegument proteins associate with the nucleocapsid, participating in microtubule-dependent cellular molecular movement, specifically the transport of the nucleocapsid by the cytoplasmic dynein/dynactin complex (step 3; Granzow et al., 2005; Maurer et al., 2008), which transports the nucleocapsid to the nuclear pore and releases viral DNA into the nucleus with the assistance of various viral proteins (step 4; Batterson et al., 1983; Ojala et al., 2000; Copeland et al., 2009). The cyclization of linear double-stranded DNA molecules into the nucleus activates the transcription and translation of viral IE genes under the action of transcriptional activators (such as VP16; step 5; Batterson and Roizman, 1983), and the translated IE proteins can transactivate the transcription and translation of early genes. The translated early proteins (mainly enzymes related to viral DNA replication), in turn, activate the replication, transcription, and translation of the late genes, and the translated late proteins (mainly the structural proteins of the virus, including cortical proteins, membrane proteins, and capsid proteins; steps 6–8; Kelly et al., 2009; Melchjorsen et al., 2009). In the nucleus, the replicated viral DNA is wrapped by capsid protein to form the nuclear capsid (step 9), and newly formed nucleocapsids bud at the inner nuclear membrane, creating primary enveloped virions within the perinuclear space. These primary envelopes then fuse with the outer nuclear membrane, allowing the nucleocapsids to be translocated into the cytoplasm (steps 10–12). Subsequently, the nucleocapsids acquire a set of tegument proteins in the cytoplasm and bud into vesicles originating from host organelles to gain the viral envelope (steps 13 and 14). These vesicles are then transported to the plasma membrane, where they fuse and release mature virions into the extracellular space (Mettenleiter, 2002; Lv et al., 2019; steps 15–16).

The UL14 gene and its homologs are essential for efficient viral growth. Studies have shown that a UL14 deletion virus forms very small plaques, produces fewer viruses, and exhibits a slightly delayed replication kinetics at a low multiplicity of infection in infected cells (Wada et al., 1999; Cunningham et al., 2000; Tandon and Mocarski, 2011; Brechtel et al., 2014). The impact of the UL14 gene-encoded protein on the viral replication cycle is specifically reflected in its regulation of IE gene transcription and expression, modulation of the nuclear localization of capsid assembly and genome packaging proteins, involvement in secondary envelopment, and influence on virion release.

#### 4.1.1 Regulation of the expression of the viral IE genes ICP0 and ICP4

During a productive virus infection, the tegument protein pUL48, a component of the HSV-1 virion that is a gene alpha transinducing factor (α-TIF, also known as VP16), interacts with the host cell transcription factor Oct-1 to initiate host cell RNA polymerases. Host RNA polymerase II (RNAP II) is recruited to the IE gene promoter to mediate IE viral gene transcription and produce IE proteins (Lu and Misra, 2000). Research has shown that HSV-1/DPV pUL14 enhances the nuclear localization of VP16, that HSV-1 pUL14 indirectly regulates the expression of the IE genes ICP4, and that DPV pUL14 significantly promotes the transcriptional activation of VP16 (Lu and Misra, 2000; Li et al., 2016; Zhou et al., 2021). These results suggest that pUL14 may indirectly regulate the transcription and expression of the IE genes ICP0 and ICP4 by enhancing the nuclear localization of VP16.

#### 4.1.2 Facilitation of the localization of crucial proteins involved in viral capsid assembly or DNA packaging

A-, B-and C-capsids were found in HSV-infected cells using sucrose density gradient ultracentrifugation. A-capsids are empty, lacking viral DNA and internal scaffolds (Gibson and Roizman, 1972; Sherman and Bachenheimer, 1988; Schrag et al., 1989; Booy et al., 1991). B-capsids contain some viral DNA and are considered intermediates in the assembly process. They do not directly transition into C-capsids but are crucial for the maturation of C-capsids, which are filled with viral DNA. Scaffold proteins, including VP22a, and considerably lower amounts of the UL26 gene products VP21 and VP24 play a role in the structural organization of B-capsids but are not merely precursors for C-capsids (Liu and Roizman, 1991, 1993; Newcomb and Brown, 1991); C-capsids contain the viral genome and are further assembled into infectious virions (Heming et al., 2017; Freeman et al., 2021). The B-type nucleocapsid was purified and analyzed using cryo-electron microscopy. The results revealed that the difference between the A-type nucleocapsid and B-type nucleocapsid particles was that the B-type capsid contained the ICP35 protein, a product of the UL26.5 gene (Newcomb et al., 1993). The capsid scaffold protein ICP35 is a capsid structural protein that temporarily operates within the capsid during viral assembly (Thomsen et al., 1995; Li X. et al., 2021). In infected Vero cells, a specific fluorescence signal of HSV-2 pUL14 was observed in the cytoplasm and nucleus at 6-h postinfection. UL14 was mainly observed in the nucleus as fine, discrete particles colocalized with the scaffolding protein ICP35. However, at 12 h postinfection, the ICP35 protein had aggregated in the perinuclear region of the cytoplasm and was located primarily in the nucleus for assembly, and colocalization with pUL14 was no longer observed. It is speculated that pUL14 may be involved in viral capsid assembly or egress (Wada et al., 1999).

DNA cleavage/packaging is mediated by proteins encoded by the UL6, UL15, UL17, UL25, UL28, UL32, and UL33 genes (Yu and Weller, 1998; Wills et al., 2006; Albright et al., 2015). Deleting any of these genes results in the aggregation of empty capsid and viral DNA duplicates in the nucleus, which cannot be assembled into the nucleocapsid. These genes have been shown to play important roles in viral assembly rather than capsid synthesis and DNA replication (Sherman and Bachenheimer, 1987; Stow, 2001; Yang et al., 2011; Borst et al., 2013). In the DNA assembly process of the nucleocapsid, the UL17 protein is a DNA assembly protein that plays a key role in the early stage of capsid assembly, participating in the entire process of cleavage and assembly. In the absence of the UL17 protein, DNA assembly cannot take place (Salmon et al., 1998; Thurlow et al., 2005). pUL33 acts as a terminal enzyme subunit, a component of the “molecular motor” involved in the cleavage/packaging of viral genomic DNA. The absence of pUL33 leads to the formation of only type B capsids and not type C capsids in the nucleus of infected cells, greatly reducing the infectivity of the virus (Al-Kobaisi et al., 1991). The UL33 protein of HSV-2 typically localizes to the cytoplasm when expressed alone and is translocated to the nucleus when co-expressed with the UL14 protein in the absence of viral infection (Yamauchi et al., 2001). These findings indicate that pUL14 can modulate the localization of pUL33; however, the exact mechanisms and effects, such as whether pUL14 is involved in DNA cleavage/packaging, should be verified.

Additionally, U2OS cells synchronously infected with UL14-deficient virus (14D) or rescued virus (14R) at an MOI of 40 PFU/cell in the presence of cycloheximide were fixed 2 h after being shifted to 37°C and permeabilized, followed by incubation with an anti-VP5 mAb for the indirect immunofluorescence analysis of the major capsid protein VP5. These 14R-infected cells showed a marked accumulation of capsids at the rim of the nucleus. In contrast, in 14D-infected cells, capsids were found at the nuclear rim to a much lesser extent. These results suggest that HSV-1 pUL14 is crucial for transporting the viral capsid protein VP5 to the nuclear rim. In addition, when HSV-2 UL14 was co-expressed with the capsid protein VP26, the cytoplasmic localization of VP26 changed to a nuclear localization (Yamauchi et al., 2001, 2008). Therefore, HSV-1/2 pUL14 can affect the localization of the capsid proteins VP5 and VP26.

#### 4.1.3 Participation in secondary envelopment

After nuclear egress, the nucleocapsid must obtain the complete set of tegument proteins and acquire its final (secondary) envelope (Mettenleiter et al., 2009; Yanagi et al., 2019). Recent studies have confirmed that the Golgi apparatus, particularly the trans-Golgi network, plays a crucial role in the envelopment of HSV (Harley et al., 2001; McMillan and Johnson, 2001), and HSV-1 pUL51 closely colocalized with Golgi marker proteins, including the Golgi-58 k protein and GM130, in transfected cells expressing only UL51 during HSV infection; however, the UL51 protein only partially colocalized with the Golgi markers. pUL51 is incorporated into virions during envelopment and localizes primarily to the inside of cytoplasmic vesicles and/or the viral envelope (Nozawa et al., 2003). Research has shown that the HSV-1 pUL51 amino acid residues Leu-111, Ile-119, and Tyr-123 are required for interaction with pUL14 in HSV-1-infected cells. In addition, in cells infected with wild-type HSV-1, 9.3% of the virus particles were unenveloped or partially enveloped nucleocapsids in the cytoplasm. However, in cells infected with ΔUL14, ΔUL51, UL51LIY/AAA, or ΔUL51/ΔUL14 viruses, 38 to 44% of the virus particles were unenveloped or partially enveloped nucleocapsids in the cytoplasm, which was 4 to 5 times greater than that in cells infected with wild-type HSV-1. These results indicated that the mutations in UL14 and/or UL51 induced comparable levels of aberrant accumulation of unenveloped capsids and partially enveloped nucleocapsids in the cytoplasm (Oda et al., 2016).

#### 4.1.4 Affects the production of virions released into cells

The nucleocapsid, after completing tegumentation and secondary envelopment in the cytoplasm, enters vesicles derived from the host membrane structure and releases virus particles from the cell (Crump, 2018). When RS cells were infected with a high MOI (5 PFU/cell) for 24 h to determine the proportion of total virus released to total virus production, the results revealed that the average release rates of an HSV-1 mutated virus lacking UL14 were 19.1 and 17.1% of the proportion of virus released into the medium by the parent and the UL14-reverted virus, respectively (Cunningham et al., 2000). In addition, Human foreskin-derived fibroblast (HF) cells were infected with HCMV UL96DDBAC (HCMV ΔUL96 BAC was also engineered to insert an FKBP-DD tag at the C-terminus) or parental virus at an MOI of 3.0 and incubated for 4 days in the presence of the encapsidation inhibitor BDCRB (20 μM) to synchronize infection. The BDCRB block was then removed, and the cells were incubated for 2 days. Analysis of cell-associated and released virus infectivity revealed a delay in UL96DDBAC virus replication compared to the parental virus (Tandon and Mocarski, 2011). These results indicate that HSV-1 UL14 or HCMV UL96 affect a virus maturation step involving efficient accumulation of cell-associated virus preceding release.

### 4.2 Maintaining cell homeostasis

pUL14 likely contributes to maintaining cellular homeostasis, such as cytoskeletal organization, after HSV-1 infection. To investigate whether pUL14 could alter the distribution of cytoskeletal proteins, immunofluorescence analysis with phalloidin and an anti-α-tubulin antibody was used to detect filamentous actin (F-actin) and microtubules in cells infected with an HSV-1 UL14-defective virus or UL14R. The results revealed that infection with the HSV-1 virus with the UL14 gene deletion led to cytoskeletal tissue destruction earlier than that observed in wild-type infected cells, suggesting that pUL14 plays a positive role in maintaining the cytoskeletal morphology of infected cells (Ohta et al., 2011).

### 4.3 pUL14 and neurovirulence

pUL14 is related to the neurotropic properties of herpesvirus. In mice intracranially injected with HSV-1 UL14-deficient viruses, the 50% lethal dose of the mutant virus was reduced by more than 30,000-fold. Additionally, recovery of the mutant virus from latently infected sacral ganglia in mice injected peripherally was significantly lower than that of the wild-type virus (Cunningham et al., 2000). In mice infected with the MHV-68 ORF35_stop_ virus, lytic replication in the lung after intranasal infection and the frequency of *ex vivo* reactivation from latency after intraperitoneal infection were lower in ORF35_stop_ virus-infected mice than in mice infected with the WT or marker-reverted virus (Hikita et al., 2015). These results indicate that UL14 plays an important role in establishing or reactivating latent infection.

### 4.4 pUL14 and apoptosis

Apoptosis, also known as programmed cell death, is one of the innate defense strategies of host cells against the invasion of foreign microorganisms such as bacteria or viruses. The replication and transmission of the virus depend on the host cell system; therefore, the host cell inhibits the proliferation and transmission of the virus through apoptosis. The virus has also evolved corresponding strategies to regulate the signaling pathway of host cell apoptosis to maintain the survival of virus-infected cells, thus facilitating viral proliferation and spread. Herpesviruses can inhibit cell apoptosis at multiple stages of viral infection to prevent premature cell death and ensure viral proliferation (Goodkin et al., 2004; Nguyen and Blaho, 2006). BHV-1, HSV-1, and HSV-2 pUL14 contain an amino acid sequence homologous to a portion of the peptide-binding domain conserved in the Hsp70 family. Various stress conditions, such as increased temperature, ATP depletion, and hyperosmotic shock, trigger the accumulation of UL14 protein in the nucleus. BHV-1 UL14 can inhibit sorbitol-induced apoptosis. Cell lines expressing HSV-2 and HSV-1 pUL14 have a greater ability to inhibit apoptosis than parental cell lines under osmotic shock and treatment with certain drugs (Yamauchi et al., 2002a, 2002b, 2003; De Martino et al., 2007). These findings indicate that pUL14 is closely related to apoptosis and that the mechanism by which pUL14 inhibits apoptosis may be related to its structural similarity to heat shock proteins.

## 5 Interaction with other host proteins

A high-throughput yeast two-hybrid system was used to identify the cellular factors that interact with UL14, which identified the potential interacting protein TSC22D3. The IFA results revealed that pUL14 and TSC22D3 were colocalized in the nucleus, whereas TSC22D3, when expressed alone, was present throughout the cell. The Co-IP results reveal that pUL14 can interact with TSC22D3 (D’Adamio et al., 1997; Wu et al., 2011). TSC22D3, also known as glucocorticoid-induced leucine zipper (GILZ), is a protein of the β-transforming growth factor-stimulated protein 22 (TSC-22) family (D’Adamio et al., 1997). Structurally, the GILZ gene consists of three domains: an N-terminal domain that includes the TSC box, a leucine zipper, and a C-terminal region that is rich in proline and glutamic acid. As a multifunctional protein, TSC22D3 is involved in interactions with many signaling molecules, including Raf, Ras, AP-1, and NF-KB, thereby influencing downstream signaling pathways (Mittelstadt and Ashwell, 2001; Marco et al., 2006). When stimulated by glucocorticoids (GCs), the glucocorticoid receptor (GR) in the cytoplasm is activated and subsequently translocated to the nucleus, where it binds to the glucocorticoid response element (GRE) on DNA, inducing the synthesis of TSC22D3 and regulating other signaling pathways (Ayroldi and Riccardi, 2009). Previous results indicate that HSV-1 can remain latent in the trigeminal ganglia and reactivate upon stimulation. During this process, glucocorticoid levels are modulated, inducing the expression of TSC22D3, which plays a regulatory role (Bener et al., 2007). These results suggest that TSC22D3 is involved in various signaling pathways and may be influenced by glucocorticoids, but the molecular mechanism of the interaction between TSC22D3 and pUL14 needs further study.

## 6 Future prospects

**Figure 4:**
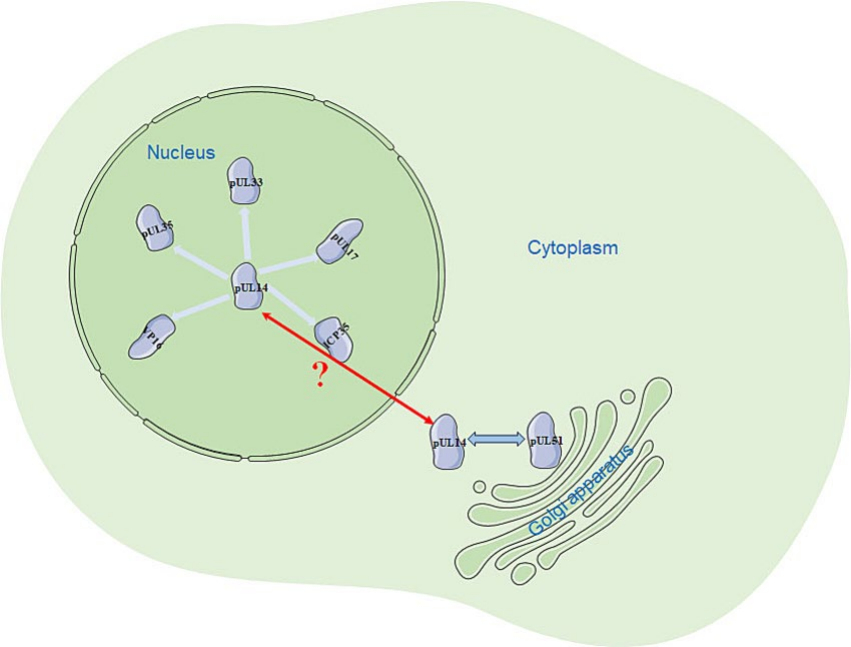
Proteins related to pUL14 subcellular localization.

Currently, studies on the herpesvirus UL14 gene have focused mainly on human herpesviruses. Studying the UL14 gene in various herpesviruses is beneficial for obtaining a more comprehensive understanding and elucidation of the functions of the UL14 gene. The UL14 gene and its homologs can present diverse subcellular localizations. In the nucleus, they can colocalize with pUL17, pUL33, pUL35, VP16, and ICP35; in the cytoplasm, they can also interact with pUL51 (Figure 4). The predicted molecular structure of this protein suggests that it may possess a potential NLS, which is due to the presence of several arginine (R)-rich regions in its N-terminus, as this basic amino acid-rich region is likely to mediate nuclear input (Lee et al., 2023). pUL14 may also have a nuclear export signal (NES; Zhou et al., 2023) that mediates protein nuclear export. Further research is needed to understand how the NLS and NES mediate the nuclear import and export of pUL14, whether other nuclear import and export receptors (such as importin α/β) are involved, and whether this process depends on Ran-GTP. The differences in the subcellular localization of pUL14 observed under transfection and viral infection conditions may be due to the influence of other viral proteins on the localization of pUL14 after viral infection. Therefore, investigating the causes behind the various localizations of pUL14 and its impact on viral processes such as budding, packaging, envelopment, and release, which are related to viral replication, can provide valuable insights into the viral replication cycle. Additionally, this research will aid in understanding the pathogenic mechanisms of herpesviruses.
